# Reappraisal of the incidence, various types and risk factors of malignancies in patients with dermatomyositis and polymyositis in Taiwan

**DOI:** 10.1038/s41598-021-83729-5

**Published:** 2021-02-25

**Authors:** Jung-Lung Hsu, Ming-Feng Liao, Chun-Che Chu, Hung-Chou Kuo, Rong-Kuo Lyu, Hong-Shiu Chang, Chiung-Mei Chen, Yih-Ru Wu, Kuo-Hsuan Chang, Yi-Ching Weng, Chun-Wei Chang, Hsing-I. Chiang, Chih-Kuang Cheng, Pai-Wei Lee, Chin-Chang Huang, Long-Sun Ro

**Affiliations:** 1 Department of Neurology, New Taipei Municipal TuCheng Hospital, Chang Gung Memorial Hospital and Chang Gung University, New Taipei City, Taiwan; 2 Department of Neurology, Chang Gung Memorial Hospital Linkou Medical Center and College of Medicine, Chang-Gung University, Linkou, Taoyuan Taiwan; 3 Graduate Institute of Mind Brain and Consciousness, Taipei Medical University, Taipei, Taiwan; 4 Brain and Consciousness Research Center, Shuang Ho Hospital, New Taipei City, Taiwan; 5 Center for Big Data Analytics and Statistics, Chang Gung Memorial Hospital, Linkou, Taoyuan, Taiwan

**Keywords:** Neuromuscular disease, Muscle, Epidemiology

## Abstract

Our study aimed to investigate the incidence, risk factors and time to occurrence of malignancy in patients with dermatomyositis (DM) and polymyositis (PM). The electronic medical records of 1100 patients with DM and 1164 patients with PM were studied between January 2001 and May 2019. Malignancies after myositis were diagnosed in 61 (5.55%) patients with DM and 38 (3.26%) patients with PM. The cumulative incidence of malignancies in patients with DM were significantly higher than patients with PM (hazard ratio = 1.78, log-rank p = 0.004). Patients with DM had a greater risk of developing malignancy than those with PM at 40–59 years old (p = 0.01). Most malignancies occurred within 1 year after the initial diagnosis of DM (n = 35; 57.38%). Nasopharyngeal cancer (NPC) was the most common type of malignancy in patients with DM (22.95%), followed by lung, and breast cancers. In patients with PM, colorectal, lung and hepatic malignancies were the top three types of malignancy. The risk factors for malignancy included old age (≥ 45 years old) and low serum levels of creatine phosphokinase (CPK) for patients with DM and male sex and low serum levels of CPK for patients with PM. Low serum levels of CPK in patients with myositis with malignancy represented a low degree of muscle destruction/inflammation, which might be attributed to activation of the PD-L1 pathway by tumor cells, thus inducing T-cell dysfunction mediating immune responses in myofibers. A treatment and follow-up algorithm should explore the occurrence of malignancy in different tissues and organs and suggested annual follow-ups for at least 5.5 years to cover the 80% cumulative incidence of malignancy in patients with DM and PM.

## Introduction

The risk factors for malignancy in patients with DM and PM included old age, male sex, cutaneous lesions, clinical characteristics and laboratory markers^15^. Regarding the type of malignancy, previous studies have shown that lung, ovarian, colorectal, bladder, breast and nasopharyngeal cancers as well as lymphoma are associated with DM, while lung, kidney, breast, bladder, endometrial, cervical, thyroid, and brain cancers as well as lymphoma are associated with PM^16^. Interestingly, studies from different countries and continents have revealed that different ethnic populations with myositis may be at risk for different malignancies, which indicates that there may be an ethnic effect in these diseases^15^. For example, nasopharyngeal carcinoma (NPC) showed a significantly higher frequency in Asian patients with DM than in European and North American patients with DM^17^. In addition, the frequency of malignancy in the general population during different study periods may affect the incidence of myositis-associated malignancy. From 1997 to 2016, the most frequent malignancies changed from uterine cervical cancer and breast cancer to breast cancer and colorectal cancer in Taiwan^18^. In the current study, we aimed to explore myositis-associated malignancy in a tertiary hospital and compare the results with those of previous studies in Taiwan.

## Materials and methods

### Subjects

We conducted a registry analysis using electronic medical records (EMRs) from the outpatient clinic and admission data from a tertiary medical center (the largest hospital in Taiwan) from January 2001 to May 2019. EMRs included demographic data, dates of clinical visits, diagnostic codes, and details of examinations. This study was based in part on data from the Chang Gung Research Database provided by Chang Gung Memorial Hospital. Subjects below 18 years of age were excluded. Diagnoses of DM and PM were based on the Bohan and Peter diagnostic criteria^19^. The study protocol was approved by the Chang Gung Memorial Hospital Institutional Review Board (CGMHIRB201901511B0). The Chang Gung Memorial Hospital Institutional Review Board approved the waiver of the participants' consent. All methods were performed in accordance with the relevant guidelines and regulations.

### Diagnostic coding

Enrollees with DM with the International Classification of Diseases (ICD), ninth revision (ICD-9) code 7103 and ICD, tenth revision (ICD-10) codes M33, M3310, M3319, M3390, M3390, M3399, and M360 or PM (or ICD-9 code 7104; or ICD-10 codes M3320 or M3329) were recruited. Amyopathic DM and inclusion body myositis were not identified due to a lack of specific diagnostic codes. Malignancies that were first diagnosed after the onset of myositis were included to assess the time-to-occurrence of cancer and malignancy risk factors after the diagnosis of myositis. Patients with a diagnosis of malignancy with the following codes were enrolled: malignant neoplasm of the lip, oral cavity or pharynx (ICD-9: 141, 145, 146, and 148; ICD-10: C02, C06, C09, C10, and C13); malignant neoplasm of the nasopharynx (ICD-9: 147; ICD-10: C11); malignant neoplasm of the esophagus (ICD-9: 150; ICD-10: C15); malignant neoplasm of the stomach (ICD-9: 151; ICD-10: C16); malignant neoplasm of the colon (ICD-9: 153; ICD-10: C18); malignant neoplasm of the rectum (ICD-9: 154; ICD-10: C20); malignant neoplasm of the liver and intrahepatic bile ducts (ICD-9: 155; ICD-10: C22); malignant neoplasm of other or unspecified parts of the biliary tract (ICD-9: 156; ICD-10: C24); malignant neoplasm of the larynx (ICD-9: 161; ICD-10: C32); malignant neoplasm of the bronchus and lung (ICD-9: 162; ICD-10: C34); secondary and unspecified malignant neoplasm of lymph nodes or leukemia (ICD-9: 169 and 196; ICD-10: C42 and C77); malignant neoplasm of the breast (ICD-9: 174; ICD-10: C50); malignant neoplasm of the corpus uteri (ICD-9: 182; ICD-10: C54); malignant neoplasm of the ovary (ICD-9: 183; ICD-10: C56); malignant neoplasm of the prostate (ICD-9: 185; ICD-10: C61); malignant neoplasm of the bladder (ICD-9: 188; ICD-10: C67); and malignant neoplasm without specification of site (ICD-9: 199; ICD-10: C80). To study the malignant neoplasms associated with comorbidities, we selected diabetes mellitus (ICD-9: 250; ICD-10: I110, I101, I109, I110, I111, and I119), hypertension (ICD-9: 401 and 402; ICD-10: I10-I16), and idiopathic interstitial lung disease (ICD-9: 515 and516; ICD-10:J 841, J842, J849, and J8410)^20^.

### Statistical analysis

The demographic data of the study population were first analyzed. We examined the ratios among groups with DM, PM and specific types of malignancy. Crude malignancy incidences in patients with DM or PM were calculated as follows: the number of malignant cases that occurred among DM or PM patients divided by the total number of patients with DM or PM within the predefined periods. To assess the effect of age on the relative risk of malignancy, we stratified patients aged 20–39 years, 40–59 years, 60–79 years, and > 80 years at the time of myositis diagnosis and estimated the malignancy frequencies in patients with DM and PM. The type of malignancy, age, sex and mean interval of malignancy onset after myositis diagnosis were documented for further comparisons. We divided the follow-up periods according to half-year intervals and recorded the case number and cumulative incidence of total malignancies within each interval for patients with DM and PM. Annual reports of the different types of malignancy incidences were obtained from the Taiwan National Cancer Registry (TNCR) for comparison.

All statistical analyses were performed using SPSS (version 21.0; IBM, New York, USA). Continuous variables are expressed as the means ± standard deviations. Categorical variables are presented as numbers and ratios. Nonparametric Mann–Whitney U tests were performed to compare the mean onset age and plasma creatine phosphokinase (CPK) levels between groups. The CPK levels were determined upon diagnosis of myositis. Serum tumor markers such as carcinoembryonic antigen (CEA) and alpha fetoprotein (AFP) and autoimmune markers such as anti-nuclear antibody (ANA) and anti-double stranded DNA antibody (anti-dsDNA) and rheumatic factor (RF) were classified as positive or negative in patients with or without malignancy. Chi-squared tests and Fisher’s exact tests were used to compare myositis patients with and without malignancy in terms of age, sex, tumor markers, autoimmune markers and comorbidities. Kaplan–Meier analysis was used to compare the cumulative incidence probability of malignancy between patients with DM and PM. A Cox regression model was used to examine the hazard ratios of risk factors in patients with DM and PM. Statistical significance was defined as P < 0.05.

## Results

### Baseline characteristics

During the period of 2001–2019, we identified a total of 1100 patients with DM, of whom 68 patients diagnosed with malignancy prior to DM were excluded. A total of 1164 patients with PM were identified, and 38 patients diagnosed with malignancy prior to PM were excluded. The mean ± standard deviation of the age at diagnosis of patients with DM and PM was 49.11 ± 15.47 years and 48.53 ± 15.03 years, respectively. Among DM patients, 395 (35.91%) were male and 705 (64.09%) were female. There were 463 (39.77%) male and 701 (60.23%) female patients in the PM group (Table 1).

**Table 1:** Total and malignancy cases of DM and PM in the study population (2001–2019 May), by age and gender group.

Age group (years)	DM (N = 1100)				PM (N = 1164)				p value*
	Total		Malignancy		Total		Malignancy		
	Total	M:F	Total	M:F	Total	M:F	Total	M:F	
19	12	5:7	0	0:0	5	0:5	0	0:0	1
20–39	296	101:195	5	1:4	318	127:191	8	5:3	0.48
40–59	520	182:338	30	15:15	552	198:354	15	11:4	0.008
60–79	238	90:148	23	11:12	273	127:146	15	9:6	0.07
≥ 80	34	17:17	3	1:2	16	11:5	0	0:0	0.54
All	1100	395:705	61	28:33	1164	463:701	38	25:13	0.008

*DM* dermatomyositis, *PM* polymyositis, *M* male, *F* female; *p value: Chi-square or Fisher exact test between total DM and PM with or without malignancy.

### The occurrence of malignancies in DM and PM patients

**Figure 1:**
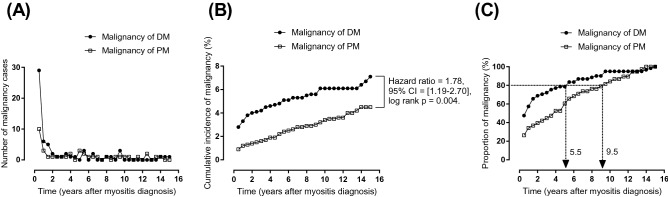
Case number (**A**), cumulative incidence (**B**) and the proportion of malignancy (**C**) in patients with DM and PM after initial diagnosis are plotted against the follow-up period in half-year intervals. The cumulative incidence showed a significant difference between patients with DM and PM based on Kaplan–Meier analysis (hazard ratio = 1.78, log-rank p value = 0.004).

**Figure 2:**
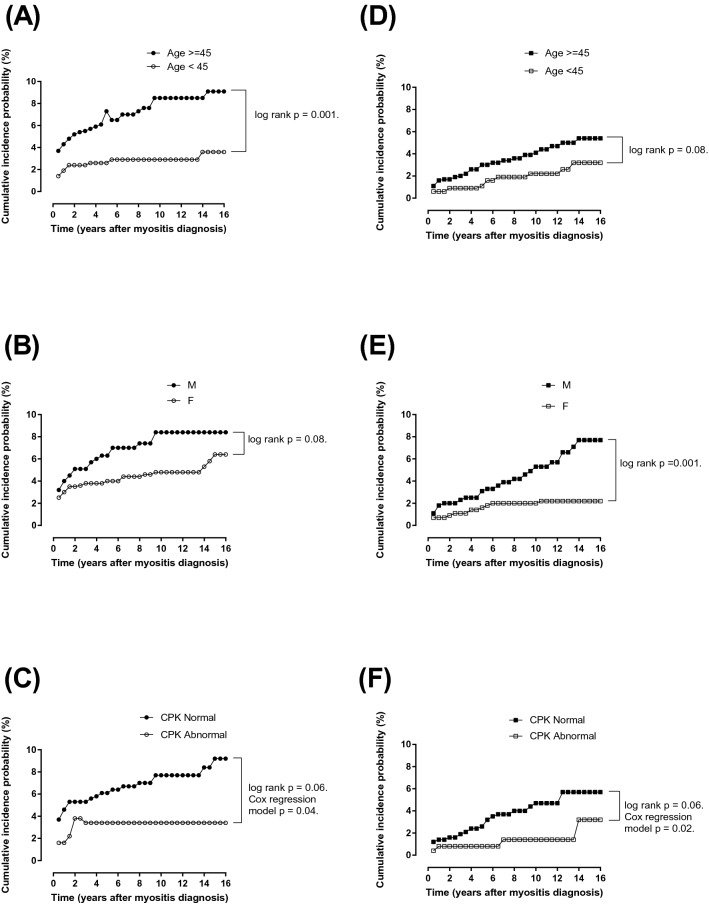
Kaplan–Meier analysis of the malignancy cumulative incidence probability in patients with DM and PM stratified by age, sex and CPK results. Panels (**A**)–(**C**) show the malignancy cumulative incidence probability stratified by age, sex and CPK results in patients with DM. Age (≥ 45 years old) showed a significantly higher malignancy incidence (log rank p = 0.001). Panels (**D**)–(**F**) show the malignancy cumulative incidence probability stratified by age, sex and CPK results in patients with PM. Male patients had a significantly higher malignancy incidence (log rank p = 0.001). Although the CPK results did not show significant differences based on Kaplan–Meier analyses in patients with DM and PM, there were significant group differences in multivariate Cox regression models (all p < 0.05).

Malignancy was diagnosed in 61 (5.55%) patients with DM and 38 (3.26%) patients with PM. According to the chi-square test, patients with DM had a significantly higher malignancy incidence than those with PM (p = 0.008). Regarding the age-stratified malignancy incidence, patients with DM had a greater risk of developing malignancy than those with PM at 40–59 years old (p = 0.01). In other age ranges, no significant differences were found. Figure 1A shows the interval between the diagnosis of DM or PM and the occurrence of malignancy. Most diagnoses of malignancy were made within 1 year after the initial diagnosis of DM (n = 35; 57.38%). However, it took 5 years after the initial diagnosis of PM for more than 50% of the diagnoses of malignancy to be made within a predefined period (n = 20; 52.63%). Figure 1B demonstrates the cumulative incidence of malignancy according to the follow-up periods, and also shows a significant difference between patients with DM and PM based on Kaplan–Meier analysis (hazard ratio = 1.78, 95% confidence interval = 1.19–2.70, log-rank p value = 0.004). Figure 1C shows the proportion of malignancy in patients with DM and PM. The curve of overall malignancy rapidly reached a plateau after 5 years in patients with DM, but it showed a progressively increasing incidence in patients with PM even after 5 years. Using the chi-square test, we divided cases of malignancy into those occurring within 1 year and those occurring after 1 year. Within 1 year of the initial diagnosis of myositis, the incidence of malignancy was significantly higher in patients with DM than in those with PM (p = 0.003). No significant difference was found in malignancy occurrence between DM and PM beyond 1 year of initial diagnosis. Figure 2 shows the Kaplan–Meier analysis of the malignancy cumulative incidence probability in patients with DM and PM stratified by age, sex and CPK results.

### Types of malignancy in patients with DM and PM

Table 2 shows information on the various types of malignancy diagnosed among patients with DM and PM. Overall, nasopharyngeal cancer (NPC) was the most common malignancy (22.95%) among patients with DM and was more common in male (83.33%) patients with DM than in female patients with DM. Ten of 14 patients were diagnosed with NPC within 1 year after the initial diagnosis of DM. Eight patients had squamous cell carcinoma, five patients had poorly differentiated carcinoma and one had carcinoma not otherwise specified (NOS). Breast cancer was the most common malignancy in female patients with DM (19.35%), and five of six patients had a pathological diagnosis of ductal adenocarcinoma. Four of six patients were diagnosed with breast cancer within 1 year after the initial diagnosis of DM. One patient with DM had two malignancies (hepatoma and prostate cancer). Among patients with PM, malignancy of the colon and rectum (15.78%) was the most common malignancy type, and four of six patients had a pathological diagnosis of adenocarcinoma. Only two patients had a diagnosis of colorectal cancer within 1 year after the initial diagnosis of PM, but four patients had a mean interval of approximately 9 years (range 58–148 months) between the initial diagnosis of PM and the occurrence of cancer. Two patients with PM had two malignancies. One patient had colorectal cancer and cervical cancer. The other patient had bladder cancer and cancer of unknown origin.

**Table 2:** Malignancy types occurred in patients with DM and PM.

Malignancy types	DM (N = 61)			PM (N = 38)		
	Male	Female	Total	Male	Female	Total
Malignant neoplasm of lip, oral cavity and pharynx	5	1	6	3	0	3
Malignant neoplasm of nasopharynx	9	5	14	0	0	0
Malignant neoplasm of esophagus	1	0	1	1	0	1
Malignant neoplasm of stomach	2	1	3	0	1	1
Malignant neoplasm of colon and rectum	0	2	2	4	2	6
Malignant neoplasm of liver and intrahepatic bile ducts	3	2	5	3	1	4
Malignant neoplasm of other and unspecified parts of biliary tract	0	1	1	1	0	1
Malignant neoplasm of larynx	0	0	0	1	0	1
Malignant neoplasm of bronchus and lung	4	3	7	3	1	4
Secondary and unspecified malignant neoplasm of lymph nodes and leukemia	1	4	5	1	3	4
Malignant neoplasm of breast	0	6	6	0	2	2
Malignant neoplasm of corpus uteri	0	2	2	0	0	0
Malignant neoplasm of ovary	0	2	2	0	0	0
Malignant neoplasm of prostate	1	0	1	3	0	3
Malignant neoplasm of bladder	0	0	0	2	1	3
Malignant neoplasm without specification of site	1	5	6	3	2	5
Total malignant neoplasm	27	34	61	25	13	38

*DM* dermatomyositis, *PM* polymyositis.

### Risk factors for malignancy in patients with DM and PM

To study the risk factors for malignancy in patients with DM and PM, we explored age, sex, diabetes mellitus, hypertension, idiopathic interstitial lung disease, plasma CPK levels, serum tumor markers and autoimmune markers in myositis patients with and without malignancy (Table 3). In patients with DM, old age (age ≥ 45 years old) and low serum levels of CPK showed a significant difference between the malignancy group and the nonmalignancy group (p = 0.003 and p = 0.05, respectively). However, sex, diabetes mellitus, hypertension and idiopathic interstitial lung disease did not show significant differences. DM patients with malignancy showed a significantly higher proportion of abnormal AFP and ANA results than patients without malignancy (p = 0.02 and p = 0.01, respectively) based on chi-squared tests. In patients with PM, male and low serum levels of CPK were significantly different between the patients with malignancy group and without malignancy group (p = 0.001, p = 0.003, respectively). PM patients with malignancy showed a significantly higher proportion of abnormal CEA and AFP results than patients without malignancy (p = 0.03 and p = 0.00, respectively).

**Table 3:** Statistic analysis of risk factors associated with PM/DM with and without malignancy.

Variables	DM (N = 1100)			PM (N = 1164)		
	Non-malignancy	Malignancy	p values	Non-malignancy	Malignancy	p values
**Age (years old)**						
< 45	424	13	0.0025	465	11	0.13
≥ 45	615	48		661	27	
**Gender**						
Female	672	33	0.09	688	13	0.001
Male	367	28		438	25	
**Diabetes mellitus**						
No	854	53	0.44	949	28	0.13
Yes	185	8		177	10	
**Hypertension**						
No	741	48	0.27	782	24	0.52
Yes	298	13		344	14	
**IILD**						
No	972	60	0.17*	1092	36	0.30*
Yes	67	1		34	2	
**CPK**						
Abnormal	196	6	0.05	280	4	0.04
Normal	420	33		426	20	
Levels of CPK (U/L)	339.06 ± 965.50	221.00 ± 626.17	0.05^#^	540.42 ± 1394.88	431.08 ± 1734.07	0.003^#^
**CEA**						
Abnormal	23	6	0.13*	16	4	0.03*
Normal	219	26		221	12	
**AFP**						
Abnormal	8	4	0.02	5	4	0.0004*
Normal	225	21		241	9	
**ANA**						
Abnormal	143	18	0.01	131	3	0.58*
Normal	317	16		350	13	
**Anti-dsDNA**						
Abnormal	8	0	1*	15	0	1*
Normal	228	22		198	3	

*DM* dermatomyositis, *PM* polymyositis, II*L*D idiopathic interstitial lung disease, *CPK* creatine phosphokinase, *CEA* carcinoembryonic antigen, *AFP* alpha fetoprotein, *ANA* anti-nuclear antibody, *Anti-dsDNA* and anti-double stranded DNA antibody; *Fisher exact test; ^#^non-parametric Mann–Whitney U test.

In the univariate Cox regression model, old age (age ≥ 45 years old) was significantly associated with malignancy in patients with DM (hazard ratio = 2.72, p = 0.001). In patients with PM, male showed a significantly higher association with malignancy (hazard ratio = 2.86, p = 0.002). In the multivariate Cox regression model, old age (age ≥ 45 years old), male and low serum levels of CPK were significantly associated with malignancies in patients with DM (hazard ratios = 2.05, 2.24 and 2.48, respectively; all p < 0.05). In patients with PM, male and low serum levels of CPK showed significantly higher associations with malignancies (hazard ratios = 5.79 and 3.62, respectively; all p < 0.05) (Table 4).

**Table 4:** Multivariate cox regression model analysis showed malignancy risk factors in patients with DM and PM.

	DM (N = 625)		PM (N = 703)	
	Hazard ratios (95% CI)	p value	Hazard ratios (95% CI)	p value
**Age (years old)**				
≥ 45	2.05 (1.01–4.15)	0.04	1.39 (0.58–3.38)	0.46
< 45				
**Gender**				
M	2.24 (1.19–4.22)	0.01	5.79 (2.28–14.74)	0.0002
F (reference)				
**Diabetes mellitus**				
Yes	0.19 (0.03–1.41)	0.10	1.37 (0.30–6.24)	0.68
No(reference)				
**Hypertension**				
Yes	1.15 (0.39–3.34)	0.79	0.60 (0.17–2.20)	0.44
No(reference)				
**IILD**				
Yes	0.59 (0.08–4.29)	0.59	3.41 (0.45–26.11)	0.24
No(reference)				
**CPK**				
Normal	2.48 (1.03–5.96)	0.04	3.62 (1.22–10.72)	0.02
Abnormal(reference)				

*DM* dermatomyositis, *PM* polymyositis, *IILD* idiopathic interstitial lung disease, *CPK* creatine phosphokinase, *CI* confidence interval.

### Incidences of malignancy compared with those in previous studies

Table 5 shows a comparison of the crude malignancy incidences from the 2003 TNCR, 2016 TNCR, a previous study from the National Health Insurance Research Database (NHIRD) by Chen et al.^10^ and our work. Both our study and Chen’s study showed increased crude malignancy incidences compared with those in the general population. In TNCR data from 2003 to 2016, the three types of malignancy with the highest annual incidences changed from 2003 (breast, hepatic and colorectal) to 2016 (breast, colorectal and lung). During this period, increased incidences of breast, colon and rectal, prostate, esophageal, bladder, hematological and lung malignancies were found. Our results did not show a trend of increasing incidence in the above malignancies. Rather, NPC was the most frequent malignancy in our study and Chen’s study. We segregated our data into the 2001–2007 period so that the study period matched that of Chen’s study. The top three malignancies in patients with DM and PM from 2001 to 2007 were similar to those from 2001 to 2019 (Table 5). Our study showed an increased incidence of malignancy in the oral cavity in patients with DM but a decreased incidence of malignancy of the breast in patients with PM compared with Chen’s study.

**Table 5:** Crude malignancy incidence (per 100,000 person) from Taiwan National Cancer Registry (TNCR), National Health Insurance Research Database (NHIRD) and current work.

	2003 TNCR	2016 TNCR	1997–2007 Chen et al. from NHIRD		2001–2019		2001–2007	
			DM (N = 1012)	PM (N = 643)	DM (N = 1100)	PM (N = 1164)	DM(N = 571)	PM(N = 658)
Total malignancy	276.68	449.59	9387.35	5132.19	5545.45	3264.60	4553.41	1671.73
Malignant neoplasm of lip, oral cavity and pharynx	27.05	33.16	98.81		545.45	257.73		151.97
Malignant neoplasm of nasopharynx	6.67	6.45	2964.43	311.04	1272.73		1401.05	
Malignant neoplasm of esophagus	6.06	11.09	98.81		90.91	85.91	175.13	
Malignant neoplasm of stomach	15.63	15.54	98.81		272.73	85.91	350.26	
Malignant neoplasm of colon and rectum	36.44	65.31	494.07	777.60	181.82	515.46	175.13	151.97
Malignant neoplasm of liver and gall bladder	44.45	51.69	494.07	622.08	545.46	429.55	175.13	
Malignant neoplasm of larynx	2.16	3.11				85.91		
Malignant neoplasm of bronchus and lung	32.80	57.30	2173.91	777.60	636.36	343.64	525.39	303.95
Secondary and unspecified malignant neoplasm of lymph nodes and leukemia	7.80	15.75	395.26	155.52	454.55	343.64	175.13	303.95
Malignant neoplasm of breast	48.02	107.20	889.33	466.56	545.45	171.82	700.53	
Malignant neoplasm of corpus uteri	34.56	20.83	296.44	311.04	181.82		350.26	
Malignant neoplasm of ovary	7.51	12.72	197.63		181.82			
Malignant neoplasm of prostate	19.43	45.73			90.91	257.73	175.13	151.97
Malignant neoplasm of bladder	8.22	19.33	197.63	311.04		257.73		303.95
Other* (pancreas, kidney, skin, bone, brain, thyroid)			790.51	1399.69	545.45	429.55	350.26	303.95

*DM* dermatomyositis, *PM* polymyositis.

## Discussion

The current study produced three major findings. First, we found that the crude malignancy incidences in patients with DM and PM were higher than those in the general population. The cumulative incidence of malignancies in patients with DM were significantly higher than patients with PM (hazard ratio = 1.78, log-rank p = 0.004). Specifically, the malignancy incidence in patients with DM ranging from 40 to 59 years old was significantly higher than that in patients with PM of the same age (p < 0.05). Second, the interval to the occurrence of malignancy showed a significant difference between patients with DM and PM. Patients with DM showed a significantly higher rate of occurrence of malignancy within 1 year after the initial diagnosis of myositis than those with PM (p < 0.05), which was similar to previous studies^5,10^. There was a tendency for the cumulative malignancy incidence to be different between the different myositis groups (p = 0.07). It took 5.5 years and 9.5 years for more than 80% of the malignancy diagnoses to be made for patients with DM and PM, respectively. Third, different types of malignancy and risk factors were found. Patients with DM more frequently had NPC, while those with PM more frequently had colorectal cancer, lung cancer and hepatic cancer. The risk factors for malignancy in patients with DM were old age, a higher proportion of abnormal AFP and ANA results and low serum levels of CPK, while those for patients with PM were male, a higher proportion of abnormal CEA and AFP results and low serum levels of CPK.

### The incidences of different types of malignancies in myositis

Increased incidences of malignancies in myositis are a well-known issue; approximately 25% of patients with DM develop malignancy within 5 years of disease onset, which is higher than the 10–15% of patients with PM^21^. In Taiwan, the incidences of malignancies in patients with DM and PM ranged from 9.4 to 23% and from 4.4 to 8.9%, respectively^5,10,20^. Our results showed that the overall incidence of malignancy was 5.6% in DM and 3.3% in PM, and this difference may be related to our exclusion of patients with malignancy diagnosed prior to a myositis diagnosis and hospital-based selection bias. Interestingly, the types of malignancy were different between Asian and Western countries. The most frequent myositis-associated malignancies were breast and ovarian cancer in women and lung and prostate cancer in men, while pancreatic, gastric, colorectal, and bladder cancer and non-Hodgkin lymphoma were also prevalent^22^. However, in a systematic review of an Asian population that included 2518 patients with myositis, malignancy was found in 10% of patients with myositis, with NPC and lung cancer being the most common associated malignancies^17^. NPC has been found to be the most frequent malignancy associated with DM in Taiwan, South China, Malaysia, Singapore and other Asian countries^10,23–25^. However, NPC was not the most frequent malignancy associated with DM in North China, Japan or Korea^14,26,27^. The risk factors for NPC included genetic factors, viral infection and environmental and dietary factors^28^. Different pathological characteristics of NPC suggested a possible major role of the internal genetic factors (e.g., undifferentiated type was found mainly in high incidence regions, while keratinizing and nonkeratinizing types were mainly found in the lower incidence regions)^29^. Viral infection in endemic regions, such as Epstein-Barr virus (EBV) infection, may accelerate the process of carcinogenesis by promoting cellular proliferation and the loss of apoptosis^28,30–32^. Diets that include nitroso compounds and other environmental factors may promote neoplastic processes and further enhance the progression of the disease ^33,34^.

### DM has a closer association with NPC than with other malignancies in Asia

During our study period, we also recorded the total numbers of different malignancies. The top five malignancies were hepatoma (n = 37,463), colorectal cancer (n = 32,296), lung cancer (n = 26,168), breast cancer (n = 22,828) and oral cancer (n = 14,667). The total number of NPC cases was 5475. The incidences of DM in the above malignancies (total number of patients with DM/total number of malignancies per 100,000 persons) were 34.7, 15.48, 91.72, 65.71, and 61.36 for hepatoma, colorectal cancer, lung cancer, breast cancer and oral cancer, respectively. The incidence of DM in NPC was 474.88 per 100,000 persons. On the other hand, the incidences of PM in the above malignancies (total number of patients with PM/total number of malignancies per 100,000 persons) were 26.69, 40.25, 22.93, 21.90, and 27.27 for hepatoma, colorectal cancer, lung cancer, breast cancer and oral cancer, respectively. The incidence of PM in NPC was 91.34. Taken together, the above findings suggested that NPC was the most common malignancy in patients with DM. These numbers indicated that there was an almost 5–15 times higher incidence in NPC than other malignancies in myositis, which demonstrated a strong association between DM and NPC.

### Possible associations between NPC and DM

NPC has been suggested to be an EBV-associated malignancy^35,36^. One study collected 172 NPC patients with and without DM and explored the EBV status. They used the two most widely tested EBV-related antibodies, IgA against early antigen antibody (EA-IgA) and IgA against viral capsid antigen (VCA-IgA), which have been used to assist with the diagnosis and prediction of the prognosis of NPC^37,38^. They found that the NPC group with DM had higher EBV VCA-IgA titers than the group without DM (p = 0.017)^32^. Another study showed significantly higher EBV genome positivity in patients with PM/DM with NPC than in those without malignancy (odds ratio = 43.9, p < 0.01), which indicated positive associations of EBV with PM/DM and NPC^39^. In addition, EBV has been associated with many autoimmune diseases^40^. Although the etiology of DM is still unknown, these findings might suggest an association between EBV infection and DM.

Recent advances in the understanding of the programmed death-1 (PD-1) checkpoint pathway in cancer and autoimmunity might help us understand the role of EBV in NPC and myositis^41,42^. The PD-1 pathway is considered a physiologic immune regulatory pathway that enables tolerance to self-antigens via the downregulation of the immune response. In previous studies, approximately 70% of EBV-positive NPC patients expressed programmed death-ligand 1 (PD-L1), and patients with DM with malignancy had higher levels of soluble PD-L1 in serum than nonmalignancy patients, which might provide evidence that EBV facilitates NPC cell production of PD-L1 and decreases local and systemic immune reactions^43,44^. In addition, persistent exposure to antigens in myositis may lead to T cell exhaustion and senescence, which might be related to the PD-1/PD-L1 pathway^45^. Less effective T cells have been shown to invade skeletal muscle tissue and contribute to a lower degree of inflammation in myofibers^45^. From the above evidence, we hypothesized that patients with NPC, DM and EBV infection might have less muscle inflammation than those without malignancy because of the activation of the PD-L1 pathway and T cell dysfunction. This hypothesis would be supported by lower serum levels of CPK, which is one of the muscle enzymes, in patients with NPC, DM and EBV infection than in those without malignancy. In our study, patients with DM associated with malignancy had significantly lower serum levels of CPK than those without malignancy (p < 0.05). Although previous studies showed that both increased and decreased levels of CPK could be risk factors for malignancy in myositis^20,46–49^, our findings suggested that lower serum levels of CPK were associated with myositis with malignancy, and this finding might be attributed to effects on the PD-L1 pathway and T cell senescence. Regarding the survival of patients with myositis and malignancy, some studies showed decreased survival of patients with DM with malignancy compared with those without malignancy^49–51^. Although we do not know the survival difference between patients with DM with and without NPC, patients with NPC with DM did not have significantly different survival from those without DM^32^.

### Risk factors for malignancy in myositis

We identified several risk factors associated with malignancy in myositis. Regarding demographic factors, old age played a significant role in malignancy associated with DM, while male contributed to malignancy associated with PM. In previous studies, some researchers found an increased incidence of malignancy in males^23,46^, while others did not find similar results^20,26^. In our study, patients with PM had colorectal, lung and prostate cancers, which are more common in male than female patients in our national cancer registry data. In terms of comorbidities, we did not find significant associations among diabetes mellitus, hypertension and interstitial lung disease and myositis-associated malignancy in our study, which is in agreement with a previous study^20^. Another study showed that a past history of diabetes mellitus was associated with an increased risk of malignancy in patients with myositis, which may be related to insulin-like growth factors, oxidative stress and DNA damage^48^. Our study showed serum tumor markers (CEA and AFP) and autoimmune markers (ANA) were associated with malignancies in patients with myositis. However, Lim et al. found no significant associations of increased tumor markers with the occurrence of malignancies, and they suggested that tumor markers were not useful for malignancy screening for patients with myositis^52^. Although our results showed significant differences between myositis patients with malignancy and without malignancy, the numbers of malignancy cases were not large enough, and interpretations should be made very cautiously.

### Limitations

Several limitations should be addressed in the current study. First, our study was from a single center and analyzed a retrospective cohort of patients with DM and PM and malignancy with a long observational period (19 years) and thus does not represent all patients with myositis and malignancy. Although we used two nationwide cohort studies as our references and some differences were found, such as differences in malignancy incidence and risk factors, some consistent findings were found, such as NPC being the most common malignancy in DM and approximately 50% of malignancies being diagnosed within 1 year after the diagnosis of DM. Second, EMR information cannot provide detailed clinical symptoms/signs, such as dysphasia, muscle power or even cutaneous lesions, which have been reported as risk factors for malignancy^15^. The differences in the severity of disease symptoms (confounding factors), the serum CPK levels at different stages of the disease (measurement errors) and the loss of some patients to follow-up (selection bias) may also limit the generalization of our findings. Third, we did not study serum myositis-specific autoantibodies, which have been reported to be related to malignancy in myositis^53,54^. Future studies including the above data may improve the sensitivity of malignancy diagnosis. Other issues include using different strategies to screen organ-specific malignancies, such as tumor markers and conventional and modern imaging techniques, which may increase the malignancy detection rate and shorten the time between myositis diagnosis and the detection of malignancy. Recent studies suggest that tumor markers are not very useful for malignancy screening; thus, we need to develop updated guidelines to improve malignancy detection in myositis^52,55^.

## Conclusion

Our study showed evidence of increased malignancy incidences in patients with DM and PM. Most malignancies in patients with DM occur within 1 year after myositis diagnosis. Specific risk factors for malignancy in myositis include old age (age ≥ 45 years old), sex (male) and low serum CPK levels. Low serum levels of CPK in myositis associated with malignancy could be explained by the activation of the PD-L1 pathway and T cell senescence.

## Data Availability

Additional clinical data are available from laboratory studies. Please contact PWL: paiwei24@gmail.com if this information is of interest.
